# Comparative toxicity of 24 manufactured nanoparticles in human alveolar epithelial and macrophage cell lines

**DOI:** 10.1186/1743-8977-6-14

**Published:** 2009-04-30

**Authors:** Sophie Lanone, Françoise Rogerieux, Jorina Geys, Aurélie Dupont, Emmanuelle Maillot-Marechal, Jorge Boczkowski, Ghislaine Lacroix, Peter Hoet

**Affiliations:** 1 INSERM, Unité 700, Paris, France; Université Paris 7, Faculté de Médecine, site X. Bichat, Paris, France, and INSERM, Unité U955, Créteil, F-94010, France; Université Paris 12, Faculté de Médecine, Créteil, F-94010, France; 2 INERIS, Verneuil-en-Halatte, France; 3 Laboratory of Pneumology, Unit for Lung Toxicology, K.U. Leuven, Herestraat 49 O&N1 bus 706, 3000 Leuven, Belgium; 4 Assistance Publique-Hôpitaux de Paris, Hôpital Bichat, CIC 007, Paris, France

## Abstract

**Background:**

A critical issue with nanomaterials is the clear understanding of their potential toxicity. We evaluated the toxic effect of 24 nanoparticles of similar equivalent spherical diameter and various elemental compositions on 2 human pulmonary cell lines: A549 and THP-1. A secondary aim was to elaborate a generic experimental set-up that would allow the rapid screening of cytotoxic effect of nanoparticles. We therefore compared 2 cytotoxicity assays (MTT and Neutral Red) and analyzed 2 time points (3 and 24 hours) for each cell type and nanoparticle. When possible, TC50 (Toxic Concentration 50 i.e. nanoparticle concentration inducing 50% cell mortality) was calculated.

**Results:**

The use of MTT assay on THP-1 cells exposed for 24 hours appears to be the most sensitive experimental design to assess the cytotoxic effect of one nanoparticle. With this experimental set-up, Copper- and Zinc-based nanoparticles appear to be the most toxic. Titania, Alumina, Ceria and Zirconia-based nanoparticles show moderate toxicity, and no toxicity was observed for Tungsten Carbide. No correlation between cytotoxicity and equivalent spherical diameter or specific surface area was found.

**Conclusion:**

Our study clearly highlights the difference of sensitivity between cell types and cytotoxicity assays that has to be carefully taken into account when assessing nanoparticles toxicity.

## Background

Engineered nanomaterials possess astonishing physical and chemical properties, which lead to an exponential development and production worldwide . For example, titanium dioxide nanoparticles possess photocatalyst activity and are used as antibacterial coatings and in sunscreens [1]. Due to their antibacterial properties, silver nanoparticles are used as medical tools, but they are also of interest in photography, jewelry, electricity and as batteries [1]. The list of actual applications and uses for nanomaterials is already substantial, and will certainly become exponential in the future. A critical issue in this wide development and subsequent use is the essential need of knowledge on nanomaterials toxicity. Several physico-chemical parameters have been proposed to be critical determinants in nanomaterial toxicity: size, crystalline structure, chemical composition, surface area, oxidation status, ... (see [2] for review). However, no single parameter has yet been identified as being the one responsible for nanomaterial toxicity. Moreover, another important factor to take into account is the nature of the cell type studied. Indeed, each cell type has its own function and therefore may not respond the same way as another cell type after exposure to one single nanomaterial. For example, Sayes and collaborators recently demonstrate that rat lung epithelial (L2 cell line) and primary alveolar macrophages exposed to different nanosized particles (carbonyl iron, silica, zinc oxide, 90–500 nm) show different sensitivity in terms of viability and inflammatory profile [3]. Nano- and fine-sized zinc oxide particles induced the highest toxicity in lung epithelial cells only, not in macrophages that were essentially resistant to all particles. Moreover, only carbonyl iron and silica nanoparticles did induce inflammatory cytokine (MIP-2) production, by macrophages only, thus showing dissociation between toxicity and inflammatory effects of these nanomaterials. In the same line, Soto and collaborators demonstrate that macrophages (from murin or human origin) do not have the same sensitivity than human alveolar epithelial cells in response to commercially manufactured inorganic nanoparticulate materials [4].

Among all engineered nanomaterials, carbon black and titanium dioxide nanoparticles have been extensively studied in terms of cytotoxic effects on various cell types, including macrophages, lung epithelial cells, fibroblasts of human or murin origin [5-8]. Beside those two types of nanoparticles, other engineered nanomaterial cytotoxic effect has been studied, such as cobalt-, copper-, iron-, zinc-, manganese-based nanomaterials [4-6,9]. However, such studies have usually been set-up to focus on one single element (i.e. cobalt, copper, iron, ...), which could be an issue when comparing biological or toxic effects of different materials. Indeed, evaluations should be performed in the context of the same experimental set-up, which allows an efficient comparison of the experimental results and, hence, the establishment of relative toxicity indexes for the different material tested [4].

We therefore performed a study aimed to evaluate the toxic effect of 24 nanoparticles of similar equivalent spherical diameter and various elemental compositions on 2 human cell lines: A549 cell line, representative of alveolar type II cells [10] and Phorbol Myristate Acetate (PMA)-differentiated monocytes to macrophages (THP-1 cell line). These 2 cell types were chosen because they are potential targets of nanomaterials *in vivo *after inhalation [11]. A secondary aim of the study was to elaborate a generic experimental set-up that would allow the rapid screening of cytotoxic effect of nanomaterials, we compared 2 cytotoxicity assays, based on metabolic activity and membrane permeability (MTT and Neutral Red respectively), and analyzed 2 time points (3 and 24 hours) for each cell type and nanomaterial. Finally, each nanomaterial was analyzed by 2 independent laboratories, out of the 3 different laboratories participating in this study. This work was performed in the framework of Nanosafe2 European project.

## Results

### Sensitivity of the different tests

**Figure 1:**
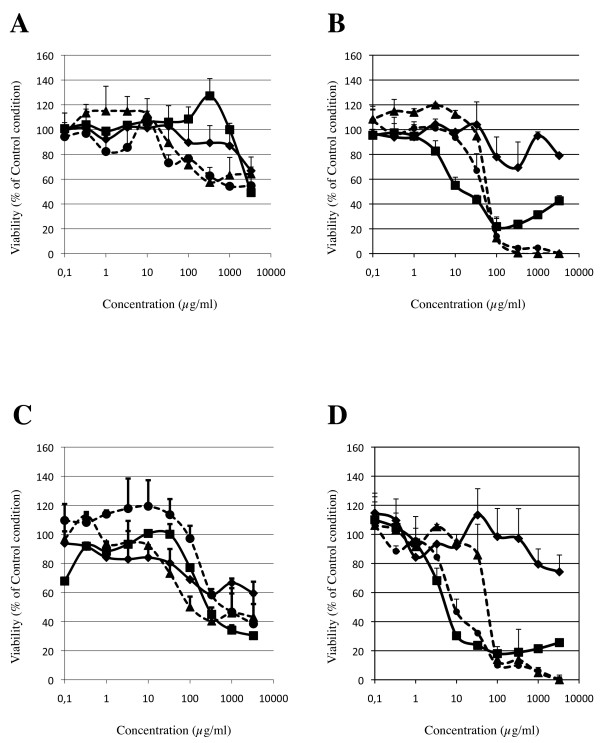
**Comparative cytotoxicities of Ceria (Panel A and C) and copperoxide (cuprous, Panel B and D) to A549 (Panel A and B) and THP-1 (Panel C and D) cells**. In each panel, values were obtained with Neutral Red assay (solid lines) after 3 hours (diamonds) and 24 hours (squares), and with MTT assay (dashed lines) after 3 hours (triangles) and 24 hours (circles).

**Figure 2:**
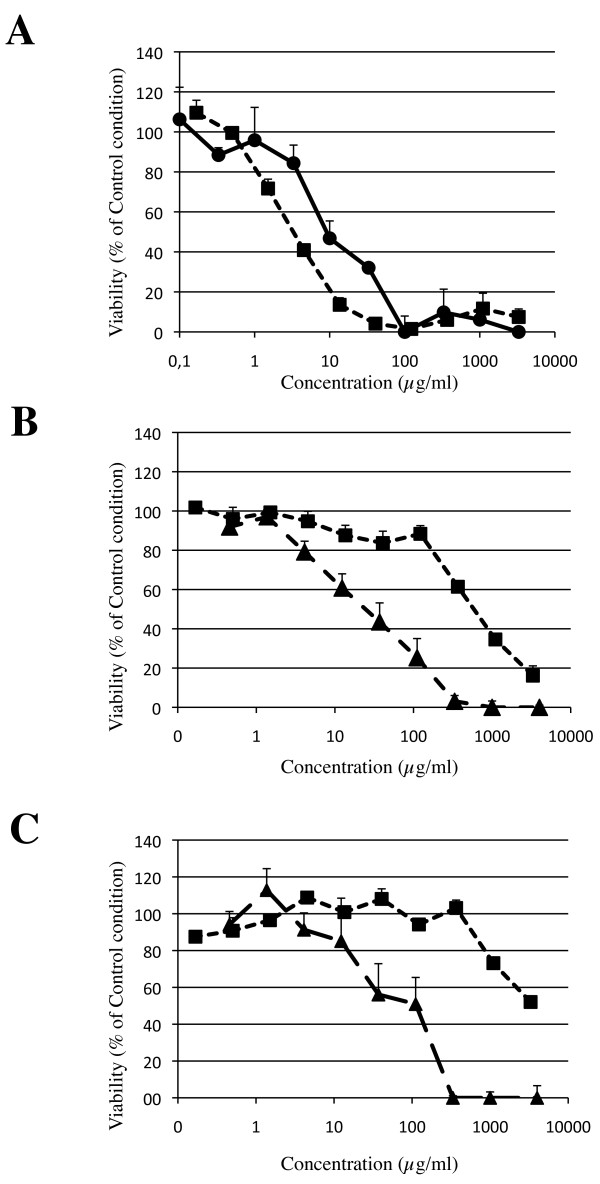
**Comparative cytotoxicities of copperoxide (Panel A), nickel oxide (Panel B) and Stainless steel (Panel C) obtained with MTT assay, after exposure of THP-1 cells for 24 hours**. In each panel, each individual lab performing the experiment is identified (Lab. A: circles, solid line, Lab. B: triangles, dashed line, Lab. C: squares, light dashed line).

Examples of toxicity curves obtained in the different experimental set-up are presented in Figure 1 and Figure 2. As described in the method section, TC50 were only calculated when at least 2 viability values were below 50% of control condition. Otherwise, the nanomaterial was considered as non-toxic in the given experimental set-up. As shown in Table 1, for each experimental set-up, the number of TC50 values that could be calculated, is higher after 24 hours than after 3 hours of incubation. Moreover, at the same time point and with the same cell type, TC50 occurrences were in higher number for MTT than for Neutral Red assay. Finally, when comparing cytotoxicity data obtained for A549 and THP-1 cells, TC50 values were obtained more often when using THP-1 cells than with A549 cells (Table 1).

**Table 1:** Number of calculated TC50 values

	**A549**				**THP-1**			
	**Neutral Red**		**MTT**		**Neutral Red**		**MTT**	

	3 hours	24 hours	3 hours	24 hours	3 hours	24 hours	3 hours	24 hours

Lab. A	1 (2.1%)	7 (14.9%)	7 (14.9%)	8 (17.0%)	5 (11.4%)	12 (26.7%)	8 (18.2%)	14 (31.1%)

Lab. B	2 (4.3%)	7 (14.9%)	6 (12.8%)	7 (14.9%)	5 (11.4%)	5 (11.1%)	13 (29.5%)	13 (28.9%)

Lab. C	2 (4.3%)	6 (12.8%)	5 (10.6%)	7 (14.9%)	9 (20.5%)	12 (26.7%)	6 (13.6%)	11 (24.4%)

Total	5 (10.6%)	20 (42.5%)	18 (38.3%)	22 (46.8%)	19 (43.2%)	29 (64.4%)	27 (61.4%)	38 (84.4%)

Number of TC50 values that could be calculated for each lab (i.e. at least 2 viability values below 50% of control condition), in each experimental condition. Percentage calculated TC50 to total number of experiments performed by each lab is given in brackets.

A similar trend is found in each lab, as shown in Figure 2. To illustrate inter-laboratory reproducibility, typical cytotoxicity curves obtained with MTT assay after 24 hours of THP-1 cells exposure to 3 different nanomaterials are shown in Figure 2. From this figure and data reported in Table 1 and additional file 1, additional file 2, additional file 3, additional file 4, additional file 5, additional file 6 and additional file 7, it is clear that for highly toxic or not toxic materials, inter-laboratory reproducibility is good, with TC50 values very similar for toxic nanomaterials. However, these data also highlight that the reproducibility for nanomaterials with intermediate toxicity is relative low.

### Cytotoxic effects of nanomaterials

Based on results mentioned above, only the cytotoxicity data obtained with MTT assay after 24 hours of THP-1 cells exposure to the different nanomaterials are presented in Table 2 (results obtained with the other experimental conditions are presented as additional file 1, additional file 2, additional file 3, additional file 4, additional file 5, additional file 6 and additional file 7). Copper- and Zinc-based nanomaterials appear to be the most toxic of all compounds tested, with TC50 values mostly below 15 μg/ml, and at the highest dose viability reaches zero for almost all those compounds (data not shown). No influence of chemical composition (relative proportion of cuprous and cupric oxide) was observed for Copper-based nanomaterials. Copper-Zinc mixed oxide was as toxic as Copper or Zinc by itself. Titania, Alumina, Ceria, Silver, Nickel and Zirconia-based nanomaterials show low to moderate toxicity, and no toxicity was observed for Tungsten Carbide. Interestingly, exposure of THP-1 cells to Cobalt nanomaterial induced toxicity only when incorporated as a Nickel-Cobalt-Manganese mixed variants, but not as Cobalt alone. It must also be noted that Cobalt from 2 different sources didn't show similar degree of cytotoxicity. For some nanoparticles with moderate to low toxicity, such as Stainless steel, Silver- or Nickel-based ones, the different labs have different outcomes, with TC50 values differing from a factor up to 70 (Nickel oxide), or TC50 values which could be calculated only for one of the two labs (Stainless steel, Nickel).

**Table 2:** Cell viability after 24 hours incubation on THP-1 cells, measured with MTT assay

Particle Name		IC50 (μg/ml)	IC75 (μg/ml)	IC25 (μg/ml)
Copper	Lab. B	1.65 (1.52–1.8)	1.04	2.62
	
	Lab. C	6.46 (1.48–28.31)	3.84	10.9

Copper (commercial source)	Lab. A	6.59 (3.68–11.81)	2.06	21.09
	
	Lab. C	5.29 (2.03–13.81)	0.96	29.11

Copper oxide (cuprous)	Lab. A	11.53 (7.48–17.7)	4.13	32.16
	
	Lab. C	3.42 (2.41–4.86)	1.58	7.41

Copper oxide (cupric)	Lab. A	31.07 (27.54–35)	21.21	45.51
	
	Lab. B	3.89 (3.31–4.57)	1.55	9.74

Copper oxide (cupric commercial source)	Lab. B	3 (2.93–3.01)	2.88	3.13
	
	Lab. C	7.3 (6.05–8.81)	4.28	12.46

Copper-Zinc mixed oxide variants	Lab. B	10.63 (7.87–14.3)	5.65	20
	
	Lab. C	13.65 (9.97–18.6)	9.13	20.4

Zinc oxide stoechiometric	Lab. A	1.66 (1.38–2)	1.19	2.33
	
	Lab. B	4.05 (3.35–4.89)	2.95	5.54

Zinc-Titania mixed oxide variants 50-50 mix	Lab. A	11.4 (8.54–15.23)	8.1	16.04
	
	Lab. C	12.8 (12.43–13.07)	11.9	13.77

Titania stoechiometric	Lab. B	432 (103.2–1809)	255.77	729.66
	
	Lab. C	NA		

Titania non-stoechiometric	Lab. A	845.2 (233.7–3056)	343.46	2079.9
	
	Lab. C	369.2 (141.2–965.4)	165.67	822.76

Silver	Lab. A	19.33 (13.8–27.09)	11.33	32.97
	
	Lab. B	NA		

Silver (commercial source)	Lab. A	1408 (379.2–5231)	162.02	>3300
	
	Lab. C	55.6 (14.98–206.3)	20.74	149.06

Cobalt	Lab. A	NT		
	
	Lab. C	NT		

Cobalt (commercial source)	Lab. A	69.6 (31.85–152.1)	43.07	112.47
	
	Lab. B	1.42 (0.48–4.17)	0.19	10.47

Nickel-Cobalt-Manganese mixed variants	Lab. A	69.13 (37.29–128.2)	20.53	232.74
	
	Lab. C	43.33 (18.31–102.5)	27.48	68.33

Nickel	Lab. B	79.46 (33.33–189.4)	21.39	295.15
	
	Lab. C	NT		

Nickel oxide	Lab. B	23.31 (17.14–31.69)	6.44	84.42
	
	Lab. C	1613 (694.6–3745)	628.18	>3300

Zirconia	Lab. A	570.6 (170.2–1913)	22.84	>3300
	
	Lab. C	171.9 (87.95–336.1)	60.07	491.87

Yttria doped Zirconia	Lab. B	107.9 (30.5–381.9)	49.45	235.44
	
	Lab. C	NA		

Stainless steel	Lab. B	62.42 (33.99–114.6)	23.32	167.05
	
	Lab. C	NT		

Alumina	Lab. A	866 (291.9–2569)	48.08	>3300
	
	Lab. B	82.19 (10.28–67.4)	13.54	498.9

Tin oxide	Lab. A	174.1 (40.48–748.3)	4	>3300
	
	Lab. B	3.39 (1.79–6.44)	1.08	10.66

Tungsten carbide	Lab. A	NT		
	
	Lab. B	NT		

Ceria	Lab. A	1058 (374.8–2984)	311.25	>3300
	
	Lab. B	NT		

TC50, TC25 and TC75 values (μg/ml) obtained with MTT assay, after 24 hours exposure of THP-1 cells, for each laboratory. 95% confidence interval is given in brackets for TC50. NT stands for Non Toxic (no TC50 could be calculated), and NA for Not Available (experiment not performed).

**Figure 3:**
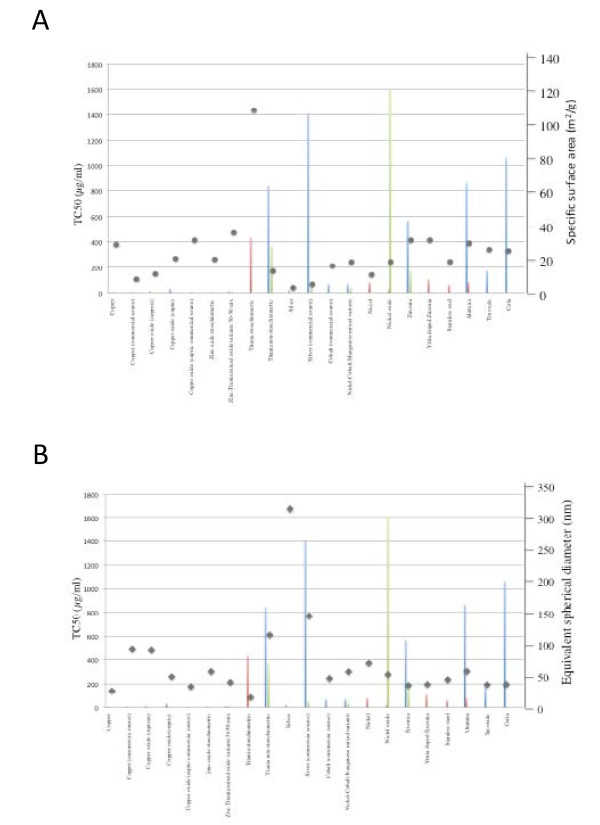
**TC50 values plotted as a function of specific surface area (squares, Panel A) or equivalent spherical diameter (Diamonds, Panel B)**. Lab. A: blue bars, Lab. B: red bars, Lab. C: green bars.

As specific surface area is often proposed as an important physical determinant of cytotoxicity, we plotted cytotoxicity data (mean of TC50 values obtained from both laboratories) against specific surface area (Figure 3A) or equivalent spherical diameter (Figure 3B) of each nanomaterial (except when the values were not concordant – NT for one lab and a calculable TC50 for the other). From Figure 3, it is apparent that there is no correlation between toxicity and either specific surface area or equivalent spherical diameter.

## Discussion

A large number of reported studies give some insights regarding cytotoxicity induced by several nanomaterials [4-9]. However, because these data are, for the most part, not obtained in the context of the same experimental set-up, it is difficult to compare with other cytotoxicity results, thus presenting an issue in the interpretation of the results. Therefore, our study was designed to evaluate and compare the toxicity induced by 24 nanoparticles, in the same experimental set-up. As expected, our results demonstrate toxicity of some, but not all, of the nanoparticles tested. Moreover, our study clearly highlights the difference of sensitivity between cell types and cytotoxicity assays that has to be carefully taken into account when assessing nanoparticle toxicity.

We found that in most cases MTT was more sensitive than Neutral Red assay to assess nanoparticle toxicity, as shown by the higher number of calculable TC50 values with MTT assay than with the Neutral Red one. Moreover, TC50 values were almost every time lower for MTT assay as compared to Neutral Red (additional file 1, additional file 2, additional file 3, additional file 4, additional file 5, additional file 6 and additional file 7). Such results are in accordance with data from literature where many examples can be found of different degrees of toxicity that could be determined for the same particle, depending on the toxicity test used [9,12-14]. This observation could be explained by the interference between the assay and the nanomaterial tested [13]. However, as described in the method section, we performed both assays carefully, (trying to avoid) making sure that no nanomaterial was present in the supernatant when reading the optical density (Neutral Red assay) or that it didn't modify the measurement (MTT assay). Another explanation probably lies in the nature of each assay, one based on the uptake and subsequent lysosomal accumulation of a supravital dye (Neutral Red assay), and the other mainly based on the metabolic activity of the mitochondria (MTT assay). As the cellular targets are not the same, one can expect the cellular answer not to be identical, depending on the cell death mechanism [12]. Such reasoning can also be used when comparing toxicity data obtained with A549 and THP-1 cells, where, in our experimental setting, A549 cells showed less sensitivity than THP-1 cells; TC50 values obtained with A549 cells were higher than those obtained with THP-1 cells. If such a difference in cell sensitivity is expected, those results appear in slight contradiction with those of Soto *et al*. [4] who analyzed the cytotoxic effects of several aggregated nanomaterials and, although finding a similar trend in both cell lines, A549 cells were shown to be more sensitive as compared to the THP-1 cells. However, they used naïve THP-1 cells (not PMA-activated) and evaluated cytotoxicity at only one time point (48 hours) after exposure to the different nanomaterials. Indeed, our results clearly showed that, whatever the cell type, there is an increase in the observed cytotoxicity, not only dose-dependently, but also time-dependently. Chang *et al*. [15], in a study comparing normal human fibroblasts to human epithelial tumour cells, proposed that the cytotoxicity induced by silica nanoparticles depends on the metabolic activity of the cell line. In that study, fibroblasts cells, with long doubling times, were more susceptible than epithelial tumor cells, which present shorter doubling times. In our study, we used two cell lines with similar doubling time (22.9 and 26 hours for A549 and THP-1 cells respectively, [ATCC product data sheet]). However, we used PMA-activated THP-1 cells, and it has been shown that PMA not only differentiates the monocytic THP-1 cells into macrophages, but also inhibits their proliferation [16]. Therefore, the paradigm proposed by Chang et al. [15] could apply to our study and explain the better sensitivity of THP-1 as compared to that of A549 cells. Another possibility to explain the difference of sensitivity observed between the two cell types is the function of phagocytosis that characterizes macrophages (THP-1 cells), but not alveolar epithelial cells (A549 cells). As such, PMA-differentiated THP-1 macrophages have a greater ability to take in particle aggregates through phagocytic mechanisms that would likely increase macrophage response to nanomaterials. Such higher sensitivity for macrophages has been shown in response to metals from combustion-derived particulate matter, after the evaluation of both cell metabolism and cell death [17]. The authors showed that rat alveolar macrophages (NR8383 cell line) were most sensitive to metals by nearly one order of magnitude in metal concentration, followed by the two alveolar epithelial cell lines studies (rat RLE-6TN and human A549). Further studies would be needed to clarify this point.

A secondary aim of our study was to generate a generic experimental set-up for a cytotoxicity screening of nanoparticle toxicity. In order to validate our findings, the experiments were performed, for each material, in two independent laboratories. Data reported in Table 2 and additional file 1, additional file 2, additional file 3, additional file 4, additional file 5, additional file 6 and additional file 7 clearly show that, for highly toxic nanomaterials (Copper- or Zinc-based), there is a good reproducibility between the independent labs; TC50 values are very similar. The same is true for not toxic nanomaterials (Tungsten Carbide and Cobalt). The reproducibility of the results between the two independent labs performing the experiments can however be questioned for nanomaterials with intermediate toxicity (Nickel oxide, Nickel, Stainless steel for example). This discrepancy appears although we designed a strict experimental set-up with as much defined and fixed parameters as possible. One can't however exclude individual variables (temperature of the culture room, batch of culture medium, spectrophotometer sensitivity, ...) that could explain the discrepancies that we observed at least for nanomaterials with intermediate toxicity. We are conscious that although care was given to be as superposable as possible, the 3 labs implied in this study couldn't be exactly the same. From Figure 2, it is clear that a rather slight shift of the cytotoxicity curve, although presenting a similar slope, makes a huge difference in the final outcome (calculated TC50 value). It can therefore be considered as quite logical that materials with intermediate toxicity differ the most when analyzed by 2 separate labs. Interestingly, we also observed that each lab presents an individual sensitivity, assessed by the values of TC50 that could be calculated; values for Lab. A are mostly higher than the 2 other labs, and Lab. B gave the lowest TC50 values. Such discrepancies, although not explained, could play a part in the differences observed for nanomaterials with intermediate toxicity.

It is difficult to compare our results with data from literature, as, as stated before, the experimental set-up is critical and therefore, relative toxicity indexes can't be defined with results obtained from different studies. Our results indicate that, out of all nanoparticles studied, Copper- and Zinc-based nanomaterials present the highest toxicity, whatever their oxidation status. The high toxicity observed for Zn-based nanomaterials is concordant with results obtained in a recent study by Park *et al*. [6] on A549 cells exposed to various inhalable metal nanoparticles. Indeed, they found that, out of 6 different nanoparticles, 100 nm Zn nanoparticles were the most cytotoxic to A549 cells, as assess by DNA fragmentation and apoptosis experiments. Interestingly, there was no uptake of Zn particles, and no change in cell morphology, the mechanism of toxicity remaining unknown [6]. In the same study, toxicity induced by Ni nanoparticles was also evaluated, and the authors demonstrated a similar increase in DNA fragmentation for Ni nanoparticles as compared to Zn nanoparticles. This is different from our results, where Zn-based nanoparticles showed higher cytotoxicity for both cell types. However, in the study by Park, there is no chemical analysis of the nanomaterial tested, and the equivalent spherical diameter is about twice that of the particles used in our study. Finally, as mentioned earlier, this discrepancy could be explained by the evaluation of different parameters (DNA fragmentation versus mitochondrial metabolism).

Physico-chemical characteristics of nanoparticles (such as size, chemical composition, crystalline structure, surface properties, ...) are proposed to be critical determinants of their toxic potential [9,18]. In the present study, we failed to show any correlation between the cytotoxicity induced by each nanoparticle, assessed by TC50 values, and its equivalent spherical diameter or specific surface area. Surface area is the physico-chemical parameter usually proposed to represent at best the specific toxicity of nanoparticles, with a good correlation between the particle surface area and the inflammatory response of animal exposed to the nanoparticles [19-22]. However, several studies also failed to demonstrate such a relationship [4,23], and care must be taken when trying to associate toxic potential of nanoparticles to only one single physico-chemical parameter, as it is probably the matter of the association of several parameters. Moreover, few of the particles we used were of similar chemical composition, therefore probably weakening a potential association between their induced cytotoxicity and their equivalent spherical diameter or specific surface area. Finally, primary particle size considerations may sometime be misleading, particularly when considering the aggregation propensity of nanomaterials, particularly in a biological medium containing salts and proteins [24-26]. The discrepancies we observed in nanoparticle-induced toxicity could be the result of differential penetration [6], generation of oxidative stress [27], inflammation [28], or a combination of several events that result in a particular toxicity mechanism. More studies are clearly needed to have a comprehensive understanding of nanoparticle-induced toxicity.

## Conclusion

As a conclusion, the work presented here allowed to efficiently compare the toxicity induced by nanomaterials differing by chemical composition, size and surface area. It isolated Cu- and Zn-based manufactured nanoparticles as nanomaterials with a potential critical use.

## Methods

### Nanomaterials

All particles were provided by QinetiQ Nanomaterials LtD, now called Intriniq Materials LtD (Farnborough, UK). The samples were distributed as part of the EU funded Framework 6 programme Nanosafe2 project and were from development batches of materials that were not fully optimized. The samples provided by QinetiQ Nanomaterials ltd thus include phase 1, phase 2 and commercially sourced powders. Particle characteristics (chemical composition, specific surface area, and equivalent spherical diameter), as provided by the supplier, are given in Table 3.

**Table 3:** Characterization of nanomaterials.

Particle Name	Chemical Composition	Morphology	Spec. Surf. Area (m^2^/g)	Equivalent Spherical Diameter (nm)
Copper	Cu_2_O (71 Wt%) CuO (22 Wt%)	Generally equiaxed particles	29.2	22.9

Copper (commercial source)	NA	Rounded agglomerated	7.18	90

Copper oxide (cuprous)	Cu_2_O	Rounded	10–11	83–94

Copper oxide (cupric)	CuO (95 Wt%) Cu_2_O (5 Wt%)	Generally equiaxed particles	20.38	45.4

Copper oxide (cupric commercial source)	NA	Rounded	32	30

Copper-Zinc mixed oxide variants	CuO (60–69 Wt%) ZnO (14–16 Wt%)	Soft agglomerates of nanopowder with some large particles	NA	NA

Zinc oxide stoechiometric	ZnO (99.5 Wt%)	Irregular	19.94	53.6

Zinc-Titania mixed oxide variants 50-50 mix	ZnO (28.8 Wt%) TiO_2 _(5.1/12.7 Wt%) Zn_2_TiO_4 _(53.5 Wt%)	Spherical/Rounded	36.7	36.7

Titania stoechiometric	Anatase TiO_2 _(99.87 Wt%)	Rounded/Spherical	114.7	12.2

Titania non-stoechiometric	Ti_4_O_7 _(39 Wt%) Ti_10_O_18 _(18 Wt%) Ti_6_O_11 _(10 Wt%) Rutile TiO_2 _(20 Wt%) Anatase TiO_2 _(13 Wt%)	Soft agglomerates of nanopowder with some large particles	12.38	112

Silver	Ag (96.3 Wt%) AgNO_2 _(3.7 Wt%)	Rounded, in necklace form	1.83	312

Silver (commercial source)	NA	Irregular	4	142

Cobalt	Co (91.49 Wt%) O (5.4 Wt%)	Rounded/Spherical	15.76	42.8

Cobalt (commercial source)	NA	Small particles necklaced together	29.2	20

Nickel-Cobalt-Manganese mixed variants	Ni (28.5 Wt%) Co (25.5 Wt%) Mn (21.2 Wt%)	Cuboid/Hexagonal	18.3	53.4

Nickel	Ni (96.99 Wt%) O (2.57 Wt%)	Rounded/Spherical	9.6–10.4	64–69

Nickel oxide	NA	Cubic	18.4	48.9

Zirconia	ZrO_2 _(>98.5 Wt%)	Rounded/Spherical	32	32

Yttria doped Zirconia	ZrO_2 _(94–96 Wt%) Y_2_O_3 _(4–6 Wt%)	Rounded/Spherical	32	33.5

Stainless steel	Fe (59.21 Wt%) Cr (20.21 Wt%) Ni (9.12 Wt%) Mo (1.02 Wt%) Si (1.01 Wt%) Mn (0.33 Wt%)	Rounded/Spherical	18.4	40.9

Alumina	Al_2_O_3_	Rounded/Spherical	24–36	40–68

Tin oxide	Sn	Irregular	26	33

Tungsten carbide	WC (46 Wt%) W (46 Wt%) WO_3 _(8 Wt%)	Mixture of spherical and angular particles	8.89	43.07

Ceria	CeO_2_/TREO (Total Rare Earth Oxide)	Rounded	25.21	33.4

Chemical composition was analyzed by Inductively Coupled plasma spectromtery ICPS/X ray fluorescence spectrometry(XRF) and Leco Combustion analysis. Particle morphology by field emission scanning electron microscopy, Specific surface area (Spec. Surf. Area) was quantified by BET method (Brunauer, Emmett and Telle), and the equivalent spherical sample Diameter calculated from the surface area measurement. The sample of nanopowder were supplied by QinetiQ Nanomaterials Ltd, now called Intriniq Materials Ltd, UK. NA = not available.

### Experimental set-up

To generate a generic experimental set-up, the toxicity of 24 different nanoparticles was assessed on 2 different cell types, alveolar cells (A549 cells) and macrophages (stimulated THP-1 cells), using 2 cytotoxicity assays: MTT and Neutral red assay. The toxic effect was analyzed at 2 time points (3 and 24 hours). For inter-laboratory comparison, each nanomaterial was analyzed by 2 independent laboratories participating in the Nanosafe2 project: K.U.Leuven (Belgium), INERIS (France), and/or INSERM (France); these labs will be assigned as Lab A, Lab B and Lab C (random order).

### Cell culture and treatment

We used human alveolar epithelial (A549) and monocyte/macrophage (THP-1) cell lines, both purchased from ATCC (Molsheim, France). In order to work in similar conditions, one single batch was purchased, and dispatched between the 3 labs (KUL, INERIS, INSERM). We defined a strict protocol for cell culture conditions, using the same cell culture media: DMEM #21969-035 and RPMI 1640 #52400-025 for A549 and THP-1 cell respectively, Invitrogen (Gibco). Both cell lines were grown in culture medium supplemented with 10% foetal bovine serum (FBS, Gibco #10106-169), 1% L-glutamine (Gibco #25030-032), 0.5% fungizone (Gibco #15290-026) and 1% penicillin-streptomycin (Gibco #15140-122). Cells were seeded in 25 cm^2 ^tissue culture flasks (#353014, BD), at 250 000 cells/flask and 900 000 cells/flask for A549 and THP-1 cells, respectively, in a total volume of 9 ml. When confluent, A549 cells were trypsinized (Trypsin-EDTA Gibco #15400-054), and seeded in 96-well plates (BD, #353072) at 30 000 cells/well (total volume 200 μl/well). THP-1 cells were centrifuged and seeded in 96-well plates at 80 000 cells/well (total volume 200 μl/well), in presence of 30 μg/ml Phorbol Myristate Acetate (PMA #P1585, Sigma-Aldrich,) in order to differentiate them into mature macrophage-like cells [16]. Twenty-four hours after seeding, cells were washed 3 times with culture medium without any additive (FBS or antibiotics), and 200 μl of particle suspension (see below) or medium alone was added to each well.

For each nanomaterial, a stock solution of 3300 μg/ml particle in culture medium without any additive was prepared, vortex at maximum speed for 1 minute and bath-sonicated for 10 minutes. One-third successive dilutions in culture medium were further performed (3300-0.1 μg/ml). Preliminary experiments demonstrated the necessity to add 0.01% Tween 80 (#P4780, Sigma) to the culture medium to obtain a homogenous suspension for Silver, Zn-Titania mixed oxide variant, Yttria-doped Zirconia and Titania stoechiometric. Cells were exposed for 3 h or 24 h to medium alone or in presence of nanomaterials. At that time, neutral red or MTT viability assays were performed (see below). Different control experiments were used to assess for interactions: 1/cells were incubated with nanomaterials (n = 2 wells per nanomaterial) with no further staining, 2/nanomaterials without cells but staining (n = 2 per nanomaterial), 3/control cells (no nanomaterial) with staining, in order to get 100% viability values (n = 6).

### Viability assays

#### Neutral Red Assay

At the end of exposure, cell culture medium was discarded, and each well washed with 200 μl Hanks Balanced Buffer Solution (HBSS+, #14025, Gibco). Cells were then incubated for 4 hours at 37°C, under 5% CO2 with 200 μl of neutral red solution. This solution was prepared as follows: Neutral red powder (#N4638, Sigma) was suspended at 0.4% in distilled water, further diluted at 1/80 in RPMI without phenol red, incubated for 24 h at 37°C, centrifuged to remove debris from neutral red powder. At that time, neutral red solution was discarded, 200 μl of formol-calcium solution (1 ml formaldehyde 40% – #415694, Carlo Erba, 10 ml CaCl2 10% – #C3881, Sigma, distilled water qsp 100 ml) was added for 1 minute, discarded, and finally 200 μl of an acid-ethanol solution (1 ml acetic acid – #45726, Sigma, plus 10 ml ethanol 50°- #20821.296, VWR) was added to each well. After 15 minutes of gentle shaking, optical density (OD) was read at 550 nm, with a spectrophotometer. Finally, in order to avoid modification of OD due to cells and/or particles, 150 μl of the supernatant of each well was transferred to a new 96-well plate and the OD read again at 550 nm. Viability was calculated as the ratio of the mean of OD obtained for each condition to that of control (no particle) condition. Values are given as means ± S.E.M.

#### MTT Assay

At the end of exposure, cell culture medium was discarded, and each well washed with 200 μl Hanks Balanced Buffer Solution (HBSS+, #14025, Gibco). Cells were then incubated for 3 hours at 37°C, under 5% CO_2 _with 200 μl of 0.5 mg/ml MTT solution (#M2128, Sigma) in HBSS. MTT solution was then discarded, and 100 μl of DiMethylSulfOxide (DMSO, #D5879, Sigma) was added to each well. Optical density was read at 550 nm, with a reference at 655 nm. Viability was calculated as the ratio of the mean of OD obtained for each condition to that of control (no particle) condition. Values are given as means ± S.E.M. In order to evaluate if any modification of OD due to particles can be measured, some OD measurement were performed again on 150 μl of the supernatant of each well that has been transferred to a new 96-well plate. No modification of OD was observed (data not shown). Therefore, all OD measurements have been performed on the original 96-wells plates.

### Statistical analysis

When at least 2 viability values were below 50% of control condition, the TC50 (toxic concentration 50, concentration of particles inducing 50% cell mortality) was calculated using GraphPad Prism software (logarithmic transformation of X-values and non linear regression -sigmoidal dose-response analysis with variable slope- with bottom and top constrains set at 0 and 100 respectively). Values are given ± 95% confidence intervals. If a TC50 could be calculated, TC25 and TC75 were calculated (respectively concentration corresponding to 75 and 25% viability), using the following equation: TCf = [(f/100-f)**1/H] * TC50 where f: percentage that needs to be calculated, H: hillslope, *: multiply, **: to the power.

## Competing interests

The authors declare that they have no competing interests.

## Authors' contributions

SL, FR, JB, GL, JG and PH designed the study. FR, JG, AD, and EMM performed the cytotoxicity assays. SL drafted the manuscript, and GL and PH helped in the final version. All authors read and approved the final manuscript.

## Acknowledgements

This study had the financial support of the European Commission through the Sixth framework programme for research and technological development NMP2-CT-2005-515843 contract "NANOSAFE2".

## Supplementary Material

Additional File 1 **cell viability after 3 hours incubation on A549 cells, measured with Neutral Red assay**. TC50, TC25 and TC75 values (μg/ml) obtained with NR assay, after 3 hours exposure of A549 cells, for each laboratory. Click here for file

Additional File 2 **cell viability after 24 hours incubation on A549 cells, measured with Neutral Red assay**. TC50, TC25 and TC75 values (μg/ml) obtained with MTT assay, after 24 hours exposure of THP-1 cells, for each laboratory. Click here for file

Additional File 3 **cell viability after 3 hours incubation on A549 cells, measured with MTT assay**. TC50, TC25 and TC75 values (μg/ml) obtained with MTT assay, after 3 hours exposure of A549 cells, for each laboratory. Click here for file

Additional File 4 **cell viability after 24 hours incubation on A549 cells, measured with MTT assay**. TC50, TC25 and TC75 values (μg/ml) obtained with MTT assay, after 24 hours exposure of A549 cells, for each laboratory. Click here for file

Additional File 5 **cell viability after 3 hours incubation on THP-1 cells, measured with Neutral red assay**. TC50, TC25 and TC75 values (μg/ml) obtained with NR assay, after 3 hours exposure of THP-1 cells, for each laboratory. Click here for file

Additional File 6 **cell viability after 24 hours incubation on THP-1 cells, measured with Neutral red assay**. TC50, TC25 and TC75 values (μg/ml) obtained with NR assay, after 24 hours exposure of THP-1 cells, for each laboratory. Click here for file

Additional File 7 **cell viability after 3 hours incubation on THP-1 cells, measured with MTT assay**. TC50, TC25 and TC75 values (μg/ml) obtained with MTT assay, after 3 hours exposure of THP-1 cells, for each laboratory. Click here for file
