# Supplementary material for: Comparative toxicity of 24 manufactured nanoparticles in human alveolar epithelial and macrophage cell lines
Source: Part Fibre Toxicol. 2009 Apr 30;6:14. doi: 10.1186/1743-8977-6-14 (PMC2685765; doi:10.1186/1743-8977-6-14)
Supplement: Additional File 1 — cell viability after 3 hours incubation on A549 cells, measured with Neutral Red assay. TC50, TC25 and TC75 values (μg/ml) obtained with NR assay, after 3 hours exposure of A549 cells, for each laboratory. [file 1743-8977-6-14-S1.docx]

**Additional Table 1:** cell viability after 3 hours incubation on A549 cells, measured with Neutral Red assay.

| Particle Name |  | IC50 (µg/ml) | IC75 (µg/ml) | IC25 (µg/ml) |
| --- | --- | --- | --- | --- |
| Copper | Lab. B | NT |  |  |
|  | Lab. C | 362.5 (72.83-1805) | 22.81 | >3300 |
| Copper (commercial source) | Lab. A | NT |  |  |
|  | Lab. C | NT |  |  |
| Copper oxide (cuprous) | Lab. A | NT |  |  |
|  | Lab. C | NT |  |  |
| Copper oxide (cupric) | Lab. A | NT |  |  |
|  | Lab. B | NT |  |  |
| Copper oxide (cupric commercial source) | Lab. B | 26.56 (15.3-51.9) | 11.3 | 79.4 |
|  | Lab. C | 2908 (304.5-2776) | 173.11 | >3300 |
| Copper-Zinc mixed oxide variants | Lab. B | NT |  |  |
|  | Lab. C | NT |  |  |
| Zinc oxide stoechiometric | Lab. A | NT |  |  |
|  | Lab. B | NT |  |  |
| Zinc-Titania mixed oxide variants 50-50 mix | Lab. A | NT |  |  |
|  | Lab. C | NT |  |  |
| Titania stoechiometric | Lab. B | NT |  |  |
|  | Lab. C | NT |  |  |
| Titania non-stoechiometric | Lab. A | NT |  |  |
|  | Lab. C | NT |  |  |
| Silver | Lab. A | 342.6 (88.67-1323) | 43.46 | 2700.61 |
|  | Lab. B | NA |  |  |
| Silver (commercial source) | Lab. A | NT |  |  |
|  | Lab. C | NT |  |  |
| Cobalt | Lab. A | NT |  |  |
|  | Lab. C | NT |  |  |
| Cobalt (commercial source) | Lab. A | NT |  |  |
|  | Lab. B | NT |  |  |
| Nickel-Cobalt-Manganese mixed variants | Lab. A | NT |  |  |
|  | Lab. C | NT |  |  |
| Nickel | Lab. B | NT |  |  |
|  | Lab. C | NT |  |  |
| Nickel oxide | Lab. B | 1481 (182.6-12012) | 74.7 | >3300 |
|  | Lab. C | NT |  |  |
| Zirconia | Lab. A | NT |  |  |
|  | Lab. C | NT |  |  |
| Yttria doped Zirconia | Lab. B | NT |  |  |
|  | Lab. C | NT |  |  |
| Stainless steel | Lab. B | NT |  |  |
|  | Lab. C | NT |  |  |
| Alumina | Lab. A | NT |  |  |
|  | Lab. B | NT |  |  |
| Tin oxide | Lab. A | NT |  |  |
|  | Lab. B | NT |  |  |
| Tungsten carbide | Lab. A | NT |  |  |
|  | Lab. B | NT |  |  |
| Ceria | Lab. A | NT |  |  |
|  | Lab. B | NT |  |  |

TC50, TC25 and TC75 values (µg/ml) obtained with NR assay, after 3 hours exposure of A549 cells, for each laboratory. 95% confidence interval is given in brackets for TC50. NT stands for Non Toxic (no TC50 could be calculated), and NA for Not Available (experiment not performed).
