# Supplementary material for: Comparative toxicity of 24 manufactured nanoparticles in human alveolar epithelial and macrophage cell lines
Source: Part Fibre Toxicol. 2009 Apr 30;6:14. doi: 10.1186/1743-8977-6-14 (PMC2685765; doi:10.1186/1743-8977-6-14)
Supplement: Additional File 2 — cell viability after 24 hours incubation on A549 cells, measured with Neutral Red assay. TC50, TC25 and TC75 values (μg/ml) obtained with MTT assay, after 24 hours exposure of THP-1 cells, for each laboratory. [file 1743-8977-6-14-S2.docx]

**Additional Table 2:** cell viability after 24 hours incubation on A549 cells, measured with Neutral Red assay.

| Particle Name |  | IC50 (µg/ml) | IC75 (µg/ml) | IC25 (µg/ml) |
| --- | --- | --- | --- | --- |
| Copper | Lab. B | 74.95 (7.39-759.7) | 18.75 | 299.63 |
|  | Lab. C | 31.27 (5.4-181.1) | 0.62 | 1566.33 |
| Copper (commercial source) | Lab. A | 1698 (155.4-1856) | 109.75 | >3300 |
|  | Lab. C | 70.79 (11.2-447.4) | 1.02 | >3300 |
| Copper oxide (cuprous) | Lab. A | 39.96 (9.33-171.1) | 2.82 | 566.94 |
|  | Lab. C | 164.7 (39.5-686) | 3.45 | >3300 |
| Copper oxide (cupric) | Lab. A | 832.7 (98.4-7046) | 10.55 | >3300 |
|  | Lab. B | 102.5 (28.23-371.9) | 32.81 | 320.19 |
| Copper oxide (cupric commercial source) | Lab. B | 11.44 (9.03-12.13) | 10.29 | 12.72 |
|  | Lab. C | 20.84 (139.4-7934) | 19.24 | >3300 |
| Copper-Zinc mixed oxide variants | Lab. B | 47.91 (15.79-145.4) | 10.27 | 223.5 |
|  | Lab. C | 54.64 (1.41-2.06) | 1.29 | 2322.3 |
| Zinc oxide stoechiometric | Lab. A | 29.12 (11.5-73.76) | 15.18 | 55.85 |
|  | Lab. B | 25.11 (13.71-46) | 19.39 | 32.51 |
| Zinc-Titania mixed oxide variants 50-50 mix | Lab. A | 92.03 (87.3-95.87) | 83.25 | 101.73 |
|  | Lab. C | NT |  |  |
| Titania stoechiometric | Lab. B | NT |  |  |
|  | Lab. C | NT |  |  |
| Titania non-stoechiometric | Lab. A | NT |  |  |
|  | Lab. C | NT |  |  |
| Silver | Lab. A | 380.9 (67.13-2161) | 61.17 | 2371.8 |
|  | Lab. B | NA |  |  |
| Silver (commercial source) | Lab. A | NT |  |  |
|  | Lab. C | NT |  |  |
| Cobalt | Lab. A | NT |  |  |
|  | Lab. C | NT |  |  |
| Cobalt (commercial source) | Lab. A | 1243 (334.1-4626) | 425.14 | >3300 |
|  | Lab. B | 241.6 (61.15-954.4) | 51.03 | 1143.7 |
| Nickel-Cobalt-Manganese mixed variants | Lab. A | NT |  |  |
|  | Lab. C | NT |  |  |
| Nickel | Lab. B | 1032 (415.1-2565) | 207.47 | >3300 |
|  | Lab. C | 509.9 (365.2-712) | 193.8 | 1341.18 |
| Nickel oxide | Lab. B | NT |  |  |
|  | Lab. C | NT |  |  |
| Zirconia | Lab. A | NT |  |  |
|  | Lab. C | NT |  |  |
| Yttria doped Zirconia | Lab. B | NT |  |  |
|  | Lab. C | NT |  |  |
| Stainless steel | Lab. B | NT |  |  |
|  | Lab. C | NT |  |  |
| Alumina | Lab. A | NT |  |  |
|  | Lab. B | NT |  |  |
| Tin oxide | Lab. A | NT |  |  |
|  | Lab. B | NT |  |  |
| Tungsten carbide | Lab. A | NT |  |  |
|  | Lab. B | NT |  |  |
| Ceria | Lab. A | NT |  |  |
|  | Lab. B | NT |  |  |

TC50, TC25 and TC75 values (µg/ml) obtained with Neutral Red assay, after 24 hours exposure of A549 cells, for each laboratory. 95% confidence interval is given in brackets for TC50. NT stands for Non Toxic (no TC50 could be calculated), and NA for Not Available (experiment not performed).
