# Supplementary material for: Comparative toxicity of 24 manufactured nanoparticles in human alveolar epithelial and macrophage cell lines
Source: Part Fibre Toxicol. 2009 Apr 30;6:14. doi: 10.1186/1743-8977-6-14 (PMC2685765; doi:10.1186/1743-8977-6-14)
Supplement: Additional File 3 — cell viability after 3 hours incubation on A549 cells, measured with MTT assay. TC50, TC25 and TC75 values (μg/ml) obtained with MTT assay, after 3 hours exposure of A549 cells, for each laboratory. [file 1743-8977-6-14-S3.docx]

**Additional Table 3:** cell viability after 3 hours incubation on A549 cells, measured with MTT assay.

| Particle Name |  | IC50 (µg/ml) | IC75 (µg/ml) | IC25 (µg/ml) |
| --- | --- | --- | --- | --- |
| Copper | Lab. B | 8.45 (1.97-36.16) | 4.96 | 14.37 |
|  | Lab. C | 123.1 (117.1-137.6) | 113.58 | 133.42 |
| Copper (commercial source) | Lab. A | 94.54 (61.3-99.8) | 85.15 | 104.97 |
|  | Lab. C | 45.18 (43.6-48.9) | 39.48 | 51.7 |
| Copper oxide (cuprous) | Lab. A | 64.4 (35.53-116.7) | 50.17 | 82.66 |
|  | Lab. C | 47.03 (35.69-61.97) | 36.43 | 60.71 |
| Copper oxide (cupric) | Lab. A | 31.79 (3.39-298) | 27.62 | 36.59 |
|  | Lab. B | 35.03 (22.6-54.29) | 24.85 | 49.39 |
| Copper oxide (cupric commercial source) | Lab. B | 15.76 (0.71-350) | 13.56 | 18.32 |
|  | Lab. C | 17.85 (1.08-294) | 15.17 | 21 |
| Copper-Zinc mixed oxide variants | Lab. B | 108.3 (34.03-344.3) | 90.02 | 130.29 |
|  | Lab. C | 388.3 (60.51-2492) | 34.94 | >3300 |
| Zinc oxide stoechiometric | Lab. A | 771.4 (171.9-3492) | 36.44 | >3300 |
|  | Lab. B | NT |  |  |
| Zinc-Titania mixed oxide variants 50-50 mix | Lab. A | NT |  |  |
|  | Lab. C | NT |  |  |
| Titania stoechiometric | Lab. B | NT |  |  |
|  | Lab. C | NT |  |  |
| Titania non-stoechiometric | Lab. A | NT |  |  |
|  | Lab. C | NT |  |  |
| Silver | Lab. A | 64.23 (39.04-105.7) | 45.58 | 90.52 |
|  | Lab. B | NA |  |  |
| Silver (commercial source) | Lab. A | NT |  |  |
|  | Lab. C | NT |  |  |
| Cobalt | Lab. A | 646.5 (225.7-1852) | 495.94 | 842.76 |
|  | Lab. C | NT |  |  |
| Cobalt (commercial source) | Lab. A | 100.4 (36.89-273.4) | 12.91 | 780.52 |
|  | Lab. B | NT |  |  |
| Nickel-Cobalt-Manganese mixed variants | Lab. A | NT |  |  |
|  | Lab. C | NT |  |  |
| Nickel | Lab. B | NT |  |  |
|  | Lab. C | NT |  |  |
| Nickel oxide | Lab. B | 805 (61.15-10597) | 62.55 | >3300 |
|  | Lab. C | NT |  |  |
| Zirconia | Lab. A | NT |  |  |
|  | Lab. C | NT |  |  |
| Yttria doped Zirconia | Lab. B | NT |  |  |
|  | Lab. C | NT |  |  |
| Stainless steel | Lab. B | NT |  |  |
|  | Lab. C | NT |  |  |
| Alumina | Lab. A | NT |  |  |
|  | Lab. B | NT |  |  |
| Tin oxide | Lab. A | NT |  |  |
|  | Lab. B | NT |  |  |
| Tungsten carbide | Lab. A | NT |  |  |
|  | Lab. B | NT |  |  |
| Ceria | Lab. A | NT |  |  |
|  | Lab. B | 440.4 (73.38-2644) | 120.73 | 1613.94 |

TC50, TC25 and TC75 values (µg/ml) obtained with MTT assay, after 3 hours exposure of A549 cells, for each laboratory. 95% confidence interval is given in brackets for TC50. NT stands for Non Toxic (no TC50 could be calculated), and NA for Not Available (experiment not performed).
