# Supplementary material for: Comparative toxicity of 24 manufactured nanoparticles in human alveolar epithelial and macrophage cell lines
Source: Part Fibre Toxicol. 2009 Apr 30;6:14. doi: 10.1186/1743-8977-6-14 (PMC2685765; doi:10.1186/1743-8977-6-14)
Supplement: Additional File 4 — cell viability after 24 hours incubation on A549 cells, measured with MTT assay. TC50, TC25 and TC75 values (μg/ml) obtained with MTT assay, after 24 hours exposure of A549 cells, for each laboratory. [file 1743-8977-6-14-S4.docx]

**Additional Table 4:** cell viability after 24 hours incubation on A549 cells, measured with MTT assay.

| Particle Name |  | IC50 (µg/ml) | IC75 (µg/ml) | IC25 (µg/ml) |
| --- | --- | --- | --- | --- |
| Copper | Lab. B | 13.49 (11.1-15.6) | 12.13 | 15 |
|  | Lab. C | 17.04 (11.51-25.24) | 11.69 | 24.84 |
| Copper (commercial source) | Lab. A | 48.13 (28.13-82.36) | 34.65 | 66.86 |
|  | Lab. C | 33.74 (28.8-42.48) | 15.54 | 68.83 |
| Copper oxide (cuprous) | Lab. A | 45 (38.6-52.45) | 26.86 | 75.39 |
|  | Lab. C | 52.52 (42.23-65.3) | 36.33 | 75.91 |
| Copper oxide (cupric) | Lab. A | 47.41 (29.65-75.83) | 34.78 | 64.62 |
|  | Lab. B | 35.61 (33.2-39.1) | 32.45 | 39.08 |
| Copper oxide (cupric commercial source) | Lab. B | 10.07 (5.71-17.73) | 6.72 | 15.08 |
|  | Lab. C | 10.82 (8.08-14.49) | 6.89 | 17 |
| Copper-Zinc mixed oxide variants | Lab. B | 14.41 (8.14-25.5) | 9.21 | 22.56 |
|  | Lab. C | 158.6 (69.72-360.8) | 29.43 | 854.57 |
| Zinc oxide stoechiometric | Lab. A | 66.17 (44.46-98.48) | 13.71 | 319.24 |
|  | Lab. B | 34.31 (32.7-36.5) | 31.99 | 36.79 |
| Zinc-Titania mixed oxide variants 50-50 mix | Lab. A | 86.73 (73.89-101.8) | 49.95 | 171.17 |
|  | Lab. C | 275.9 (203.1-374.6) | 131.53 | 578.73 |
| Titania stoechiometric | Lab. B | NT |  |  |
|  | Lab. C | NT |  |  |
| Titania non-stoechiometric | Lab. A | NT |  |  |
|  | Lab. C | NT |  |  |
| Silver | Lab. A | 42.67 (20.19-90.16) | 9.86 | 187.73 |
|  | Lab. B | NA |  |  |
| Silver (commercial source) | Lab. A | NT |  |  |
|  | Lab. C | NT |  |  |
| Cobalt | Lab. A | NT |  |  |
|  | Lab. C | NT |  |  |
| Cobalt (commercial source) | Lab. A | 1142 (405.2-3220) | 225.21 | >3300 |
|  | Lab. B | 3039 (81.72-112980) | 803.97 | >3300 |
| Nickel-Cobalt-Manganese mixed variants | Lab. A | NT |  |  |
|  | Lab. C | NT |  |  |
| Nickel | Lab. B | NT |  |  |
|  | Lab. C | NT |  |  |
| Nickel oxide | Lab. B | 994.9 (937.3-1007.9) | 935.77 | 1057.77 |
|  | Lab. C | NT |  |  |
| Zirconia | Lab. A | 911.2 (613.4-1354) | 667.02 | 1244.75 |
|  | Lab. C | 82.88 (39.99-171.8) | 34.68 | 198.07 |
| Yttria doped Zirconia | Lab. B | NT |  |  |
|  | Lab. C | NT |  |  |
| Stainless steel | Lab. B | NT |  |  |
|  | Lab. C | NT |  |  |
| Alumina | Lab. A | NT |  |  |
|  | Lab. B | NT |  |  |
| Tin oxide | Lab. A | NT |  |  |
|  | Lab. B | NT |  |  |
| Tungsten carbide | Lab. A | NT |  |  |
|  | Lab. B | NT |  |  |
| Ceria | Lab. A | NT |  |  |
|  | Lab. B | NT |  |  |

TC50, TC25 and TC75 values (µg/ml) obtained with MTT assay, after 24 hours exposure of A549 cells, for each laboratory. 95% confidence interval is given in brackets for TC50. NT stands for Non Toxic (no TC50 could be calculated), and NA for Not Available (experiment not performed).
