# Supplementary material for: Comparative toxicity of 24 manufactured nanoparticles in human alveolar epithelial and macrophage cell lines
Source: Part Fibre Toxicol. 2009 Apr 30;6:14. doi: 10.1186/1743-8977-6-14 (PMC2685765; doi:10.1186/1743-8977-6-14)
Supplement: Additional File 5 — cell viability after 3 hours incubation on THP-1 cells, measured with Neutral red assay. TC50, TC25 and TC75 values (μg/ml) obtained with NR assay, after 3 hours exposure of THP-1 cells, for each laboratory. [file 1743-8977-6-14-S5.docx]

**Additional Table 5:** cell viability after 3 hours incubation on THP-1 cells, measured with Neutral red assay.

| Particle Name |  | IC50 (µg/ml) | IC75 (µg/ml) | IC25 (µg/ml) |
| --- | --- | --- | --- | --- |
| Copper | Lab. B | 0.08 (0-16315) | 0.36 | 0.02 |
|  | Lab. C | 60.47 (25.98-140.7) | 23 | 158.92 |
| Copper (commercial source) | Lab. A | NT |  |  |
|  | Lab. C | 26.16 (12.65-54.1) | 6.63 | 103.25 |
| Copper oxide (cuprous) | Lab. A | NT |  |  |
|  | Lab. C | 47.69 (11.72-194) | 1.30 | 1746.81 |
| Copper oxide (cupric) | Lab. A | 154.6 (59.65-400.8) | 63.92 | 373.89 |
|  | Lab. B | NT |  |  |
| Copper oxide (cupric commercial source) | Lab. B | 2413 (302.4-19254) | 12.67 | >3300 |
|  | Lab. C | NT |  |  |
| Copper-Zinc mixed oxide variants | Lab. B | NT |  |  |
|  | Lab. C | 9.26 (3.41-25.16) | 1.95 | 43.95 |
| Zinc oxide stoechiometric | Lab. A | 14.11 (8.19-24.3) | 5.43 | 36.68 |
|  | Lab. B | 0.14 (0-6.4) | 0.02 | 0.79 |
| Zinc-Titania mixed oxide variants 50-50 mix | Lab. A | 354.3 (122.6-1024) | 61.52 | 2040.44 |
|  | Lab. C | 61.52 (21.87-173.1) | 3.36 | 1125.23 |
| Titania stoechiometric | Lab. B | 538.5 (225.6-1285) | 204.03 | 142125 |
|  | Lab. C | NA |  |  |
| Titania non-stoechiometric | Lab. A | 2193 (1500-3250) | 759.44 | >3300 |
|  | Lab. C | 753.7 (326.9-1738) | 249.02 | 2281.22 |
| Silver | Lab. A | 59.77 (20.89-171) | 3.44 | 1038.49 |
|  | Lab. B | NA |  |  |
| Silver (commercial source) | Lab. A | NT |  |  |
|  | Lab. C | 531.4 (704.1-12333) | 147.35 | 1916.37 |
| Cobalt | Lab. A | NT |  |  |
|  | Lab. C | NT |  |  |
| Cobalt (commercial source) | Lab. A | NT |  |  |
|  | Lab. B | NT |  |  |
| Nickel-Cobalt-Manganese mixed variants | Lab. A | NT |  |  |
|  | Lab. C | 809.9 (163.4-4014) | 196.53 | >3300 |
| Nickel | Lab. B | NT |  |  |
|  | Lab. C | NT |  |  |
| Nickel oxide | Lab. B | NT |  |  |
|  | Lab. C | NT |  |  |
| Zirconia | Lab. A | NT |  |  |
|  | Lab. C | NA |  |  |
| Yttria doped Zirconia | Lab. B | 582.1 (268.8-1261) | 162.14 | 2089.84 |
|  | Lab. C | NA |  |  |
| Stainless steel | Lab. B | NT |  |  |
|  | Lab. C | 196 (36.85-1043) | 14.08 | 2728.24 |
| Alumina | Lab. A | NT |  |  |
|  | Lab. B | NT |  |  |
| Tin oxide | Lab. A | NT |  |  |
|  | Lab. B | NT |  |  |
| Tungsten carbide | Lab. A | NT |  |  |
|  | Lab. B | NT |  |  |
| Ceria | Lab. A | NT |  |  |
|  | Lab. B | NT |  |  |

TC50, TC25 and TC75 values (µg/ml) obtained with NR assay, after 3 hours exposure of THP-1 cells, for each laboratory. 95% confidence interval is given in brackets for TC50. NT stands for Non Toxic (no TC50 could be calculated), and NA for Not Available (experiment not performed).
