# Supplementary material for: Comparative toxicity of 24 manufactured nanoparticles in human alveolar epithelial and macrophage cell lines
Source: Part Fibre Toxicol. 2009 Apr 30;6:14. doi: 10.1186/1743-8977-6-14 (PMC2685765; doi:10.1186/1743-8977-6-14)
Supplement: Additional File 6 — cell viability after 24 hours incubation on THP-1 cells, measured with Neutral red assay. TC50, TC25 and TC75 values (μg/ml) obtained with NR assay, after 24 hours exposure of THP-1 cells, for each laboratory. [file 1743-8977-6-14-S6.docx]

**Additional Table 6:** cell viability after 24 hours incubation on THP-1 cells, measured with Neutral red assay.

| Particle Name |  | IC50 (µg/ml) | IC75 (µg/ml) | IC25 (µg/ml) |
| --- | --- | --- | --- | --- |
| Copper | Lab. B | 5.7 (0.07-452.6) | 40.08 | 0.81 |
|  | Lab. C | 0.39 (0.11-1.42) | 0.03 | 5.05 |
| Copper (commercial source) | Lab. A | 114.7 (19.43-677.2) | 6.22 | 2114. 16 |
|  | Lab. C | 13.15 (1.86-92.81) | 1.41 | 122.93 |
| Copper oxide (cuprous) | Lab. A | 8.15 (2.97-22.36) | 2.44 | 27.41 |
|  | Lab. C | 2.93 (0.58-14.9) | 0.28 | 30.85 |
| Copper oxide (cupric) | Lab. A | 12.38 (3.92-39.11) | 0.94 | 163.79 |
|  | Lab. B | NT |  |  |
| Copper oxide (cupric commercial source) | Lab. B | 17.88 (15.7-19.2) | 16.31 | 19.6 |
|  | Lab. C | 4.98 (0.88-28.1) | 0.2 | 122.53 |
| Copper-Zinc mixed oxide variants | Lab. B | 1911 (457.7-7978) | 101.04 | >3300 |
|  | Lab. C | 87.91 (20.94-369.1) | 25.04 | 308.68 |
| Zinc oxide stoechiometric | Lab. A | 2.08 (1.4-3.09) | 1.11 | 3.89 |
|  | Lab. B | 81.22 (1.2-5506) | 6.02 | 1095.84 |
| Zinc-Titania mixed oxide variants 50-50 mix | Lab. A | 10 (9.1-11.5) | 9.9 | 10.1 |
|  | Lab. C | 19.74 (4.79-81.37) | 1.49 | 261.17 |
| Titania stoechiometric | Lab. B | 95.97 (20.57-447.8) | 6.15 | 1497.05 |
|  | Lab. C | NA |  |  |
| Titania non-stoechiometric | Lab. A | 1062 (605.3-1864) | 262.16 | >3300 |
|  | Lab. C | 220.5 (123.6-393.1) | 79.2 | 613.86 |
| Silver | Lab. A | 18.36 (9.94-34) | 10.66 | 31.68 |
|  | Lab. B | NA |  |  |
| Silver (commercial source) | Lab. A | NT |  |  |
|  | Lab. C | 27.6 (10.74-70.92) | 4.33 | 175.83 |
| Cobalt | Lab. A | NT |  |  |
|  | Lab. C | NT |  |  |
| Cobalt (commercial source) | Lab. A | 164.7 (26.3-1031) | 3.88 | >3300 |
|  | Lab. B | NA |  |  |
| Nickel-Cobalt-Manganese mixed variants | Lab. A | 112.9 (51.84-246) | 14.82 | 860.24 |
|  | Lab. C | 0.63 (0.08-4.6) | 0.05 | 8.05 |
| Nickel | Lab. B | NT |  |  |
|  | Lab. C | 141 (37.13-535.6) | 23.81 | 834.9 |
| Nickel oxide | Lab. B | NT |  |  |
|  | Lab. C | 2.36 (1.19-4.67) | 0.15 | 37.02 |
| Zirconia | Lab. A | NT |  |  |
|  | Lab. C | NT |  |  |
| Yttria doped Zirconia | Lab. B | NT |  |  |
|  | Lab. C | NT |  |  |
| Stainless steel | Lab. B | NT |  |  |
|  | Lab. C | 145.8 (92.03-230.9) | 45.89 | 463.21 |
| Alumina | Lab. A | 672.5 (210.2-2152) | 100.35 | >3300 |
|  | Lab. B | NT |  |  |
| Tin oxide | Lab. A | 562.1 (165.5-1909) | 9.77 | >3300 |
|  | Lab. B | NT |  |  |
| Tungsten carbide | Lab. A | NT |  |  |
|  | Lab. B | NT |  |  |
| Ceria | Lab. A | 476.6 (162.7-1396) | 101.65 | 2234.65 |
|  | Lab. B | NT |  |  |

TC50, TC25 and TC75 values (µg/ml) obtained with NR assay, after 24 hours exposure of THP-1 cells, for each laboratory. 95% confidence interval is given in brackets for TC50. NT stands for Non Toxic (no TC50 could be calculated), and NA for Not Available (experiment not performed).
