# Supplementary material for: Comparative toxicity of 24 manufactured nanoparticles in human alveolar epithelial and macrophage cell lines
Source: Part Fibre Toxicol. 2009 Apr 30;6:14. doi: 10.1186/1743-8977-6-14 (PMC2685765; doi:10.1186/1743-8977-6-14)
Supplement: Additional File 7 — cell viability after 3 hours incubation on THP-1 cells, measured with MTT assay. TC50, TC25 and TC75 values (μg/ml) obtained with MTT assay, after 3 hours exposure of THP-1 cells, for each laboratory. [file 1743-8977-6-14-S7.doc]

**Additional Table 7:** cell viability after 3 hours incubation on THP-1 cells, measured with MTT assay.

| Particle Name |  | IC50 (µg/ml) | IC75 (µg/ml) | IC25 (µg/ml) |
| --- | --- | --- | --- | --- |
| Copper | Lab. B | 5,89 (0,53-1) | 2,26 | 15,3 |
|  | Lab. C | 117,6 (63,53-217,6) | 75,19 | 183,94 |
| Copper (commercial source) | Lab. A | 30,56 (24,02-38,88) | 21,48 | 43,48 |
|  | Lab. C | 37,81 (35.4-36.8) | 33,54 | 42,62 |
| Copper oxide (cuprous) | Lab. A | 57,48 (43,43-76,08) | 40,46 | 81,67 |
|  | Lab. C | 28,16 (18,66-42,5) | 16,04 | 49,44 |
| Copper oxide (cupric) | Lab. A | 37,85 (21.5-53.8) | 1,48 | 33,55 |
|  | Lab. B | 4,94 (0,75-32,5) | 42,7 | 16,53 |
| Copper oxide (cupric commercial source) | Lab. B | 16,21 (15.23-18.51) | 13,67 | 19,22 |
|  | Lab. C | 44,64 (31,13-64,02) | 34,51 | 57,74 |
| Copper-Zinc mixed oxide variants | Lab. B | 58,15 (21,48-157,4) | 22,09 | 153,08 |
|  | Lab. C | 1562 (530,7-4598) | 278,32 | >3300 |
| Zinc oxide stoechiometric | Lab. A | 491,4 (218,8-1103) | 49,32 | >3300 |
|  | Lab. B | 22,33 (12,31-40,49) | 8,44 | 59,09 |
| Zinc-Titania mixed oxide variants 50-50 mix | Lab. A | 771,3 (435,4-1366) | 216,94 | 2742,26 |
|  | Lab. C | 263,2 (95,41-726,3) | 112,61 | 615,18 |
| Titania stoechiometric | Lab. B | 336,4 (227,3-498) | 129,95 | 870,86 |
|  | Lab. C | NA |  |  |
| Titania non-stoechiometric | Lab. A | NT |  |  |
|  | Lab. C | NT |  |  |
| Silver | Lab. A | 19,74 (10,27-37,93) | 12,35 | 31,56 |
|  | Lab. B | NA |  |  |
| Silver (commercial source) | Lab. A | NT |  |  |
|  | Lab. C | NT |  |  |
| Cobalt | Lab. A | NA |  |  |
|  | Lab. C | NA |  |  |
| Cobalt (commercial source) | Lab. A | 232,6 (27,59-1960) | 57,65 | 938,43 |
|  | Lab. B | 304,3 (297.3-327.1) | 283,68 | 326,41 |
| Nickel-Cobalt-Manganese mixed variants | Lab. A | NT |  |  |
|  | Lab. C | NT |  |  |
| Nickel | Lab. B | 170,8 (55,43-526,4) | 30,53 | 955,45 |
|  | Lab. C | NT |  |  |
| Nickel oxide | Lab. B | 45,96 (18,24-115,8) | 17,56 | 120,27 |
|  | Lab. C | NT |  |  |
| Zirconia | Lab. A | NT |  |  |
|  | Lab. C | NT |  |  |
| Yttria doped Zirconia | Lab. B | NT |  |  |
|  | Lab. C | NT |  |  |
| Stainless steel | Lab. B | 144,9 (21,72-966,5) | 41,53 | 505,6 |
|  | Lab. C | NT |  |  |
| Alumina | Lab. A | NT |  |  |
|  | Lab. B | 314,5 (301.4-345.2) | 282 | 350,75 |
| Tin oxide | Lab. A | NT |  |  |
|  | Lab. B | 22,72 (13,83-37,34) | 5,74 | 89,95 |
| Tungsten carbide | Lab. A | NT |  |  |
|  | Lab. B | NT |  |  |
| Ceria | Lab. A | 4223,1 (126,7-1413) | 31,92 | >3300 |
|  | Lab. B | 521,9 (242,4-1124) | 189,78 | 1435,24 |

TC50, TC25 and TC75 values (µg/ml) obtained with MTT assay, after 3 hours exposure of THP-1 cells, for each laboratory. 95% confidence interval is given in brackets for TC50. NT stands for Non Toxic (no TC50 could be calculated), and NA for Not Available (experiment not performed).
